# Degeneration of human photosensitive retinal ganglion cells may explain sleep and circadian rhythms disorders in Parkinson’s disease

**DOI:** 10.1186/s40478-018-0596-z

**Published:** 2018-09-10

**Authors:** Isabel Ortuño-Lizarán, Gema Esquiva, Thomas G. Beach, Geidy E. Serrano, Charles H. Adler, Pedro Lax, Nicolás Cuenca

**Affiliations:** 1 Department of Physiology, Genetics and Microbiology, University of Alicante, 03690 San Vicente del Raspeig, Spain; 2 Banner Sun Health Research Institute, Sun City, AZ 85351 USA; 3 Mayo Clinic Arizona, Scottsdale, AZ 85259 USA

**Keywords:** Retina, Parkinson’s disease, Circadian rhythms, Sleep disorders, Melanopsin retinal ganglion cell, Human

## Abstract

Parkinson’s disease (PD) patients often suffer from non-motor symptoms like sleep dysregulation, mood disturbances or circadian rhythms dysfunction. The melanopsin-containing retinal ganglion cells are involved in the control and regulation of these processes and may be affected in PD, as other retinal and visual implications have been described in the disease. Number and morphology of human melanopsin-containing retinal ganglion cells were evaluated by immunohistochemistry in eyes from donors with PD or control. The Sholl number of intersections, the number of branches, and the number of terminals from the Sholl analysis were significantly reduced in PD melanopsin ganglion cells. Also, the density of these cells significantly decreased in PD compared to controls. Degeneration and impairment of the retinal melanopsin system may affect to sleep and circadian dysfunction reported in PD pathology, and its protection or stimulation may lead to better disease prospect and global quality of life of patients.

## Introduction

The retina is an accessible and visible tissue, part of the central nervous system (CNS). Its well defined and highly characterized layered structure, together with the extensive knowledge about its neurons, synaptic contacts and physiology, make the retina an ideal material for pathophysiological studies of the CNS. In fact, neurodegenerative diseases mainly observed in the brain such as Parkinson’s disease (PD), Alzheimer’s disease, or Multiple Sclerosis present similar signs of degeneration in the retina [14], which is considered as a “window to the brain”.

Intrinsically photosensitive melanopsin-containing retinal ganglion cells (mRGCs) are, together with cones and rods, retinal photoreceptors. While cones and rods are responsible for vision forming pathways, mRGCs are also in charge of the non-image forming pathways that primarily control and measure light irradiance detection [27, 48]. Melanopsin, an opsin protein containing a vitamin A-based chromophore maximally sensitive at 479 nm [40, 47], is the photopigment contained within mRGCs. Melanopsin-containing RGCs project to different CNS regions and regulate physiological and behavioral responses as important as circadian rhythms, pupillary reflex, melatonin production or mood [28, 30].

PD is the second most common neurological disorder and affects over 10 million people worldwide (http://parkinson.org/understanding-parkinsons/causes-and-statistics). Its main motor clinical features are rigidity, tremor and bradykinesia [19, 21, 46], but people with PD may also have several non-motor symptoms including cognitive decline and dementia [11], gastrointestinal and cardiovascular problems [43], mood disturbance [50], visual disruption [2, 55], impairment of the pupillary reflex response [54], and sleep disorders [19, 46]. Sleep disorders including REM sleep behavior disorder (RBD), altered sleep, and hypersomnolence are extremely common in PD patients, affecting up to a 90% [10, 56]. Moreover, people with PD also exhibit alterations in the circadian secretion pattern of melatonin [9]. Dysfunction of circadian rhythms in PD is thought to be one of the causes of sleep disturbances and it can lead to cognitive and metabolic deficits, psychiatric and mood symptoms, or cardiovascular problems, negatively impacting quality of life [56].

The defining pathological lesions of PD are Lewy bodies and associated neurites with cytoplasmic accumulation of α-synuclein phosphorylated at serine-129 (p-α-syn), and the loss of dopaminergic neurons in the *substantia nigra pars compacta* [5, 13, 19]. The latter has traditionally been considered the cause of the motor clinical manifestations. Nevertheless, PD is today mostly considered as a multisystem disorder in which other different nervous system subdivisions are affected. Brain regions involved in vision are affected in PD, including the hypothalamic suprachiasmatic nucleus [16] and the retina [6, 45], both of which exhibit p-α-syn deposits. This visual system pathology in PD is accompanied by clinical findings including reduced electroretinography response and reduced visual evoked potentials, lower contrast sensitivity and impaired color and motion perception [3, 39]. These all suggest that vision is strongly affected at a cellular level.

As retinal mRGCs innervate the suprachiasmatic nucleus [20] and are jointly responsible for regulating circadian rhythms, which are in turn involved in mood and sleep behaviors, mRGCs dysfunction may be at least partially involved in the PD pathological process. Others have previously proposed a link between mRGCs, circadian rhythms and sleep regulation [1, 32], and a relationship between sleep disturbances and morphological impairment of mRGCs in human with aging has been described [18]. Therefore, the aim of this study was to evaluate the morphological changes of human mRGCs in PD, hypothesizing an involvement in sleep and circadian dysfunction. In this work, we show that the retinal melanopsin system is impaired in PD. We demonstrate that mRGCs degenerate in PD, as revealed by its number reduction and their morphological alterations, and this fact may be linked to the circadian and sleep disturbances suffered by PD patients.

## Materials and methods

### Human retinas

Human retinas from 11 donors were obtained postmortem, within 6 h of death, from the Arizona Study of Aging and Neurodegenerative Disorders (AZSAND), the Banner Sun Health Research Institute Brain and Body Donation Program (BBDP; http://www.brainandbodydonationprogram.org/). All procedures were in accordance with the Declaration of Helsinki and with the recommendations and protocols approved by the Ethics Committee of the University of Alicante. Signed written informed consent was provided by all the participants in the study. Human donors, both men and women, were not significantly different in age, ranging from 70 to 82 years at death, and did not report any past history of retinal diseases.

The control group consisted of patients without neurodegenerative diseases (*n* = 5) and the Parkinson’s disease group (*n* = 6) included subjects with a typical clinicopathological profile, diagnosed from the BBDP. Standard tests and neuropathological examinations were performed in deceased subjects as previously described [7].

### Retinal histology

The human enucleated eyes were fixed in formaldehyde (3,75–4%) for 2 h at room temperature or 24–72 h at 4 °C, washed in PBS and then successively cryoprotected in increasing sucrose solutions of 15%, 20% and 30%. After removing the iris, lens and vitreous body, the retina was extracted and dissected, obtaining eight quadrants. The superior-nasal portion was used for further analysis.

### Immunoperoxidase labeling

Wholemount retinas were stained using the immunoperoxidase labeling technique described by Esquiva et al. [17, 18]. Following inactivation of endogenous peroxidase activity with 1% H_2_O_2_ (H1009; Sigma, St. Louis, MO, USA), retinas were incubated in 2.28% NaIO_4_ (S1878; Sigma) and later in 0.02% NaBH_4_ (163314; Panreac, Barcelona, Spain). Then, flat-mount retinas were incubated in the anti-melanopsin primary antibody (1:5000; UF028) for 3 days at 4 °C. This antibody, raised against the 15 N-terminal amino acids of human melanopsin, was kindly provided by Dr. Ignacio Provencio (University of Virginia, Charlottesville, VA, USA). After the incubation time, they were washed in PBS, incubated for 2 days in a goat anti-rabbit biotinylated secondary antibody (1:100; 111–064-9144; Jackson ImmunoResearch Laboratories, West Grove, PA, USA), and then incubated 2 more days in an avidin-biotin peroxidase complex solution (0.9% avidin + 0.9% biotin; PK-6100, Vectastain Elite ABC Kit; Vector Laboratories Ltd., Cambridgeshire, UK). Retinas were finally washed and incubated in a fresh solution of 0.1% 3,3′-diaminobenzidine tetrahydrochloride (DAB, D5637; Sigma) plus 0.01% H_2_O_2_ and 0.025% ammonium nickel (II) sulfate hexahydrate (A1827; Sigma) until the staining was revealed as a brown precipitate. After DAB reaction, flat retinas were prepared with the ganglion cell layer side up, and coverslipped for optical microscopy (Leica DMR; Leica Microsystems).

To determine their type and morphology, immunostained mRGCs were traced by hand in all flat-mounted retinas using a camera lucida connected to a Leica DMR microscope (Leica Microsystems). Images were then digitized, using image-editing software (Adobe Photoshop 10.0; Adobe Systems, Inc., San Jose, CA, USA). Total number of cells expressing melanopsin was counted and density of mRGCs per mm^2^ was calculated.

### Morphological analysis

Representative mRGCs were traced by hand in order to recreate their soma and dendritic profiles using a camera lucida (120 cells analyzed in total, 60 cells of controls and 60 of PD, 15 cells of each morphological subtype and group).

To analyze the morphology of mRGCs the Bonfire program developed in the Firestein laboratory at Rutgers University [33] was used. From digitized neuritic arbors, this software allowed us to perform a Sholl analysis and to estimate the number of branch points, the terminal neurite tips and the total number of Sholl intersections per cell [49].

### Statistical analysis

Statistical analysis was performed using Prism 6 for Windows (Graphpad Software, Inc., La Jolla, CA, USA). To assess the differences of the studied variables, both globally or per mRGC subtype (M1, M1d, M2 and M3), between PD and control patients a non-parametric two-tailed Mann-Whitney test was used. Differences of the Sholl curve representing the number of intersections per distance between PD and controls were evaluated using a paired non-parametric Wilcoxon signed rank test. In all cases, a *p*-value lower than 0.05 was considered statistically significant.

## Results

### Types of mRGCs in the human retina

**Fig. 1:**
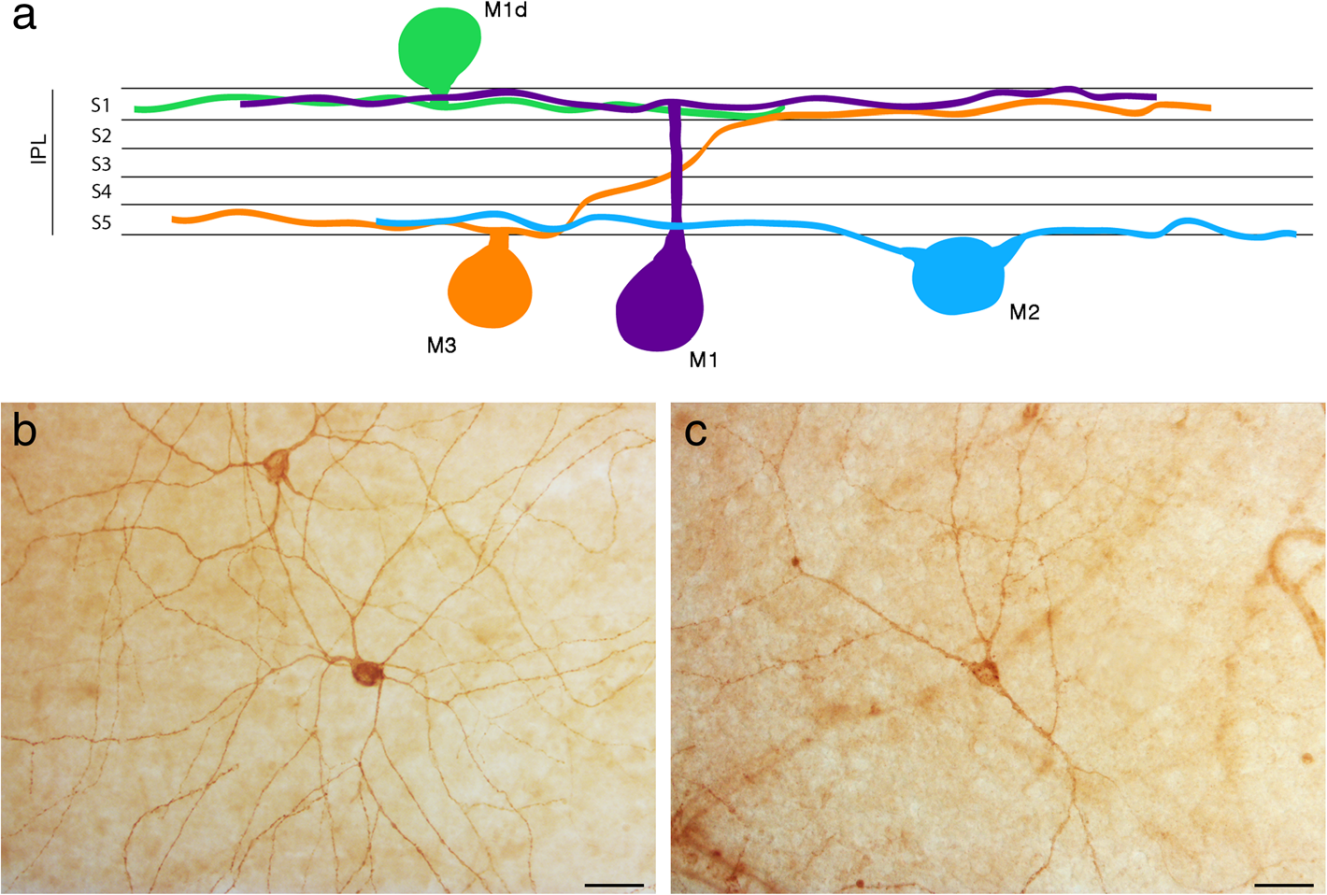
Melanopsin retinal ganglion cells in the human retina. **a** Diagram showing the structure and classification of mRGC depending on their soma location and dendrites stratification in IPL S1 or S5. **b**, **c** Immunostaining of human melanopsin using the DAB method in flat wholemount retinas of control (**b**) and PD (**c**) subjects. Scale bar, 50 μm

In the human retina, four types of mRGCs are found. They are classified in accordance with their soma location and dendritic stratifications: M1, M1d, M2, and M3. As the diagram of Fig. 1 shows, M1, M2 and M3 cells have their soma located in the ganglion cell layer, while their dendrites stratify in different strata of the inner plexiform layer (IPL). M1 cells stratify in the S1 plexus of the IPL, M2 stratify in S5 plexus, and M3 has dendrites in both strata: S1 and S5. M1d cell is a displaced M1 cell, with its soma located in the inner nuclear layer (INL) and its dendrites in the S1 plexus of the IPL, near the INL [8]. M1d mRGC is the predominant type in the human retina, accounting for about half of all mRGCs [18, 25]. An example of control and PD DAB immunostained M1d mRGC is shown in Fig. 1a and b respectively. Notice the lower dendrite complexity in PD mRGC (Fig. 1b) and its lower staining intensity when compared to controls (Fig. 1a). Identification of S1 and S5 strata can be done changing the microscope focus and using the Nomarski technique of differential interference contrast. This technique allows us to identify and differentiate the INL, the IPL, and the ganglion cell layer (GCL). Thus, S1 and S5 strata can be differentiated, without need of counterstaining, because S1 is near the INL and S5 in the opposite side of the IPL, near the GCL.

### Decrease of mRGC density in PD retinas

**Fig. 2:**
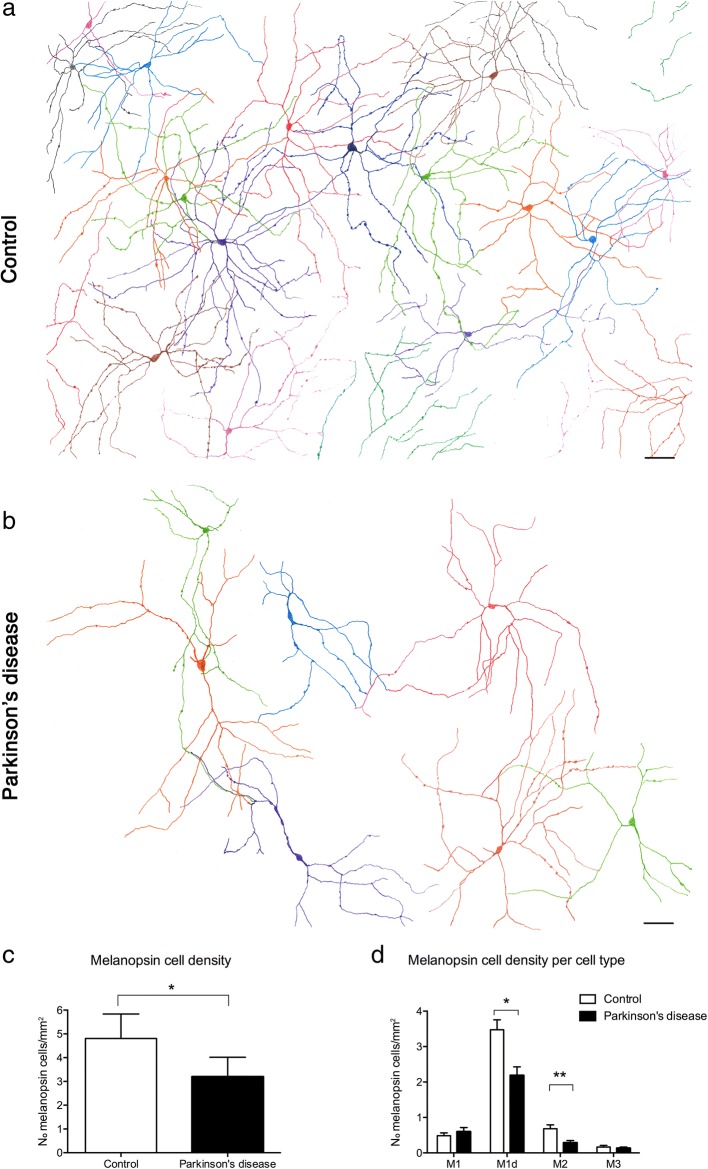
Representative drawings of control and PD retinal fields. Each color defines an individual mRGC. **a** Melanopsin plexus in a control wholemount retina. **b** Melanopsin plexus in a PD wholemount retina. **c** Total mRGC quantification (number of mRGCs per mm^2^) and comparison between control and PD subjects. **d** Comparison of the mRGC density per cell type in control and PD subjects. Scale bar, 100 μm. Data is presented as mean ± s.d. *P < 0.05, **P < 0.01

Cell density quantification and morphological analysis were performed to evaluate differences in mRGCs between PD and control subjects. These studies were made considering the total mRGCs as well as differentiating by mRGC type. A reduction in the mRGC density and in the complexity of the melanopsin plexus was found. Fig. 2a and b are drawings representing retinal fields that show mRGCs and its plexus in control and PD. Fig. 2c and d show the density of mRGCs, expressed as number of mRGCs per mm^2^, both totally and by mRGC type. A reduction in number of mRGCs, accompanied by a drastic reduction in their plexus complexity, can be clearly seen in the drawings. The decrease in mRGC number is statistically significant (control: 4.8 ± 1.3 cells/mm^2^; PD: 3.2 ± 0.8 cells/mm^2^; *p*-value = 0.05) and it mostly affects the M1d and M2 mRGC types. In normal conditions, mRGCs make contacts to other mRGCs creating a dense plexus and have numerous dendritic beads, as the control drawing shows. In PD, the plexus is highly reduced and there are very few contacts between cells and fewer dendritic beads.

### Morphological impairment of mRGCs in PD

**Fig. 3:**
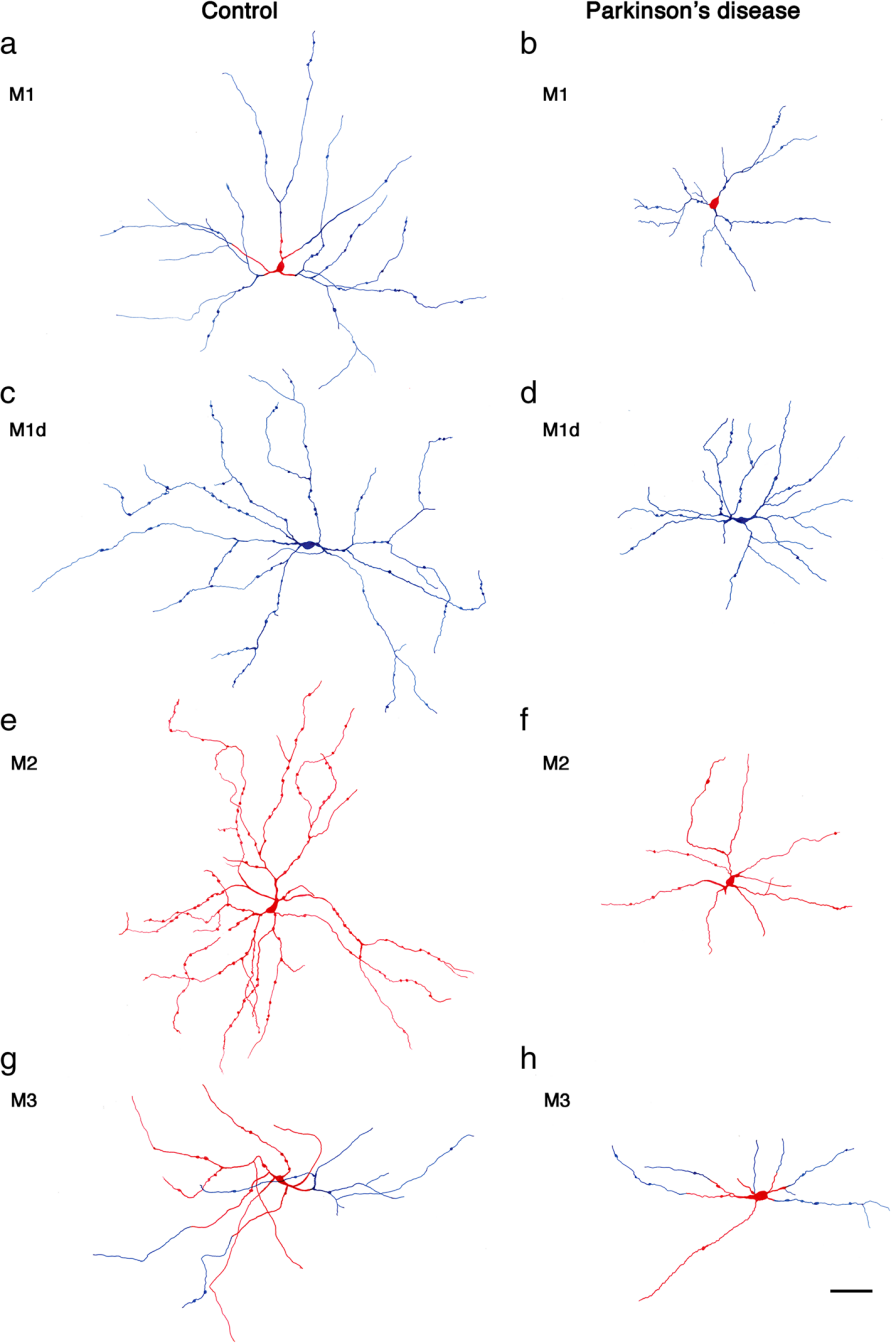
Representative drawings of control and PD mRGCs. Blue indicates the parts of the cell located in the S1 IPL layer and red shows the parts of the cell located in the S5 IPL layer. **a**, **b** M1 mRGC in control and PD respectively. **c**, **d** M1d mRGC in control and PD respectively. **e**, **f** M2 mRGC in control and PD respectively. **g**, **h** M3 mRGC in control and PD respectively. Scale bar, 100 μm

Apart from cell density decline, morphological changes were also found in PD mRGCs. Morphology, dendritic arborization and dendritic tree size of normal and PD cells can be observed in Fig. 3. Red drawings show the elements that are placed in the IPL S5 stratum and blue represent the elements from S1 stratum, allowing the differentiation of the mRGC types. Visually, structure, size and dendritic trees are altered in PD compared to controls: dendritic area is reduced and cells have fewer and shorter ramifications. Dendritic beads, which are thought to represent synaptic contact points, are also reduced in PD (control: 44.7±25.8 beads per cell; PD: 19.3±10.9 beads per cell; *p*-value < 0.0001), what could be a sign of functional alteration. These morphological alterations are drastic in M1, M1d and M2 cells, while M3 cells seem to be the least affected by the disease.

**Fig. 4:**
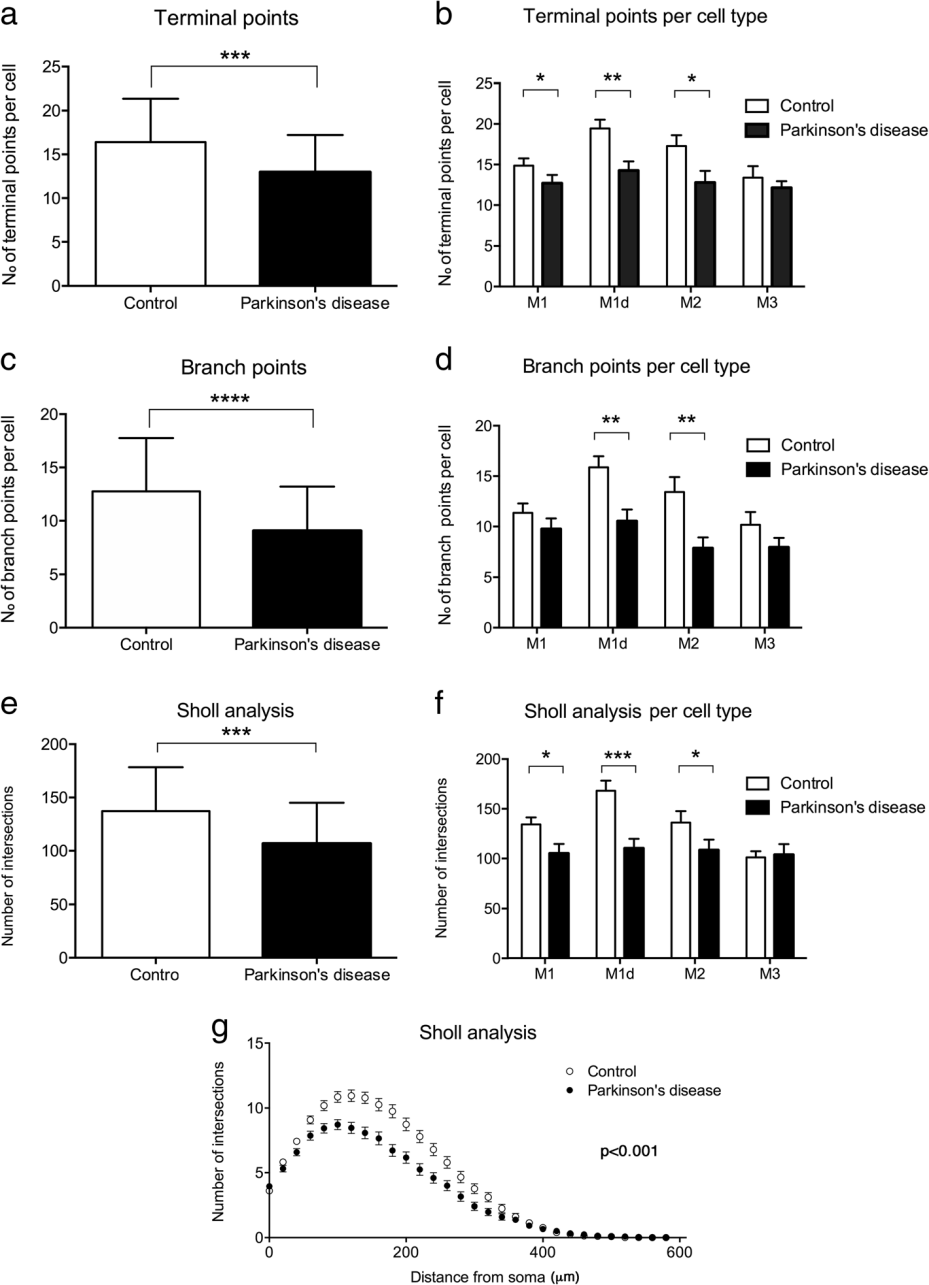
Morphological Sholl analysis of mRGCs in PD and controls. **a**, **b** Comparison of terminal points number per cell in PD and controls considering total mRGC (**a**) or per cell type (**b**). **c**, **d** Comparison of the number of branch points in all mRCGs (**c**) and per cell type (**d**) in PD and controls. **e**, **f** Sholl area comparison of PD and control mRGCs globally (**e**) and per cell type (**f**). Data is presented as mean ± s.d. *P < 0.05, **P < 0.01, ***P < 0.001, ****P < 0.0001. **g** Sholl analysis curve representing number of intersections per distance from soma, comparing total controls and PD mRGCs. Data is presented as mean ± s.e.m.

Morphological changes were measured using the Sholl analysis, that includes the total number of intersections, the number of intersections per distance, and terminal points and branch points numbers. Results are shown in Fig. 4 and corroborate the differences described above. The three analyzed measures are significantly reduced in PD, compared to controls: terminal points decrease from 16 ± 5 (controls) to 13 ± 4 (PD) (*p*-value < 0.001); branch points from 13± 5 to 9± 4 (*p*-value < 0.0001); and Sholl total number of intersections from 137,2 ± 41,3 to 106,8 ± 38,2 (*p*-value < 0.001). When comparing these parameters in each cell type, M1d and M2 cells show statistically significant reduced values in PD in the three measures, M1 cells show a statistically significant decrease in Sholl analysis and terminal points, and M3 were not significantly affected, although they present a tendency for fewer terminal and branch points. The Sholl analysis curve (Fig. 4g) shows the number of intersections per distance from the cell soma. Less intersections have been found in PD until a distance from the soma of 340 μm, what indicates a lower cell complexity and less ramifications of PD mRGCs, as concluded also by the less branches, terminal points, and number of total intersections that they present in comparison to controls.

## Discussion

In recent years, a huge effort has been made to study the state and health of brain regions related to circadian rhythms disturbances and sleep dysregulation, like the suprachiasmatic nucleus, but these alterations are not yet completely understood and some regions are found not to be affected until advanced states of the disease [22]. The study of the retinal mRGCs is a new approach in trying to understand the cellular mechanisms underlying circadian rhythms dysfunctions in PD and may add valuable information to the current knowledge of the disease.

This study demonstrates a loss of melanopsin-immunoreactive RGCs in Parkinson’s disease compared to control subjects. The density of mRGCs is significantly decreased in PD patients, and the remaining cells exhibit morphological alterations like decreased Sholl area, fewer ramifications and terminal points, and a reduced melanopsin-immunoreactive plexus. These morphological changes and numerical reduction demonstrate that mRGCs are affected in PD, probably by dying or losing melanopsin production, and it is likely that both of these would lead to functional impairment. To the best of our knowledge, this is the first study that describes alterations of mRGCs in PD.

A recent study in humans show that the mRGC density and plexus decrease with age and correlate it with the circadian rhythm dysfunction observed with aging [18]. In the present study, the mRGC type most affected is M1d, the main mRGC type in the human retina; that is also the type most affected by age [18]. M2 cells also have lower cell densities and both M1 and M2 show altered morphological parameters. These differences are not significant in the aging retina, but in PD it seems that almost all mRGCs show morphological abnormalities as well as a numerical decline. As mRGCs innervate the suprachiasmatic nucleus [20], it is expected that these morphological alterations lead to a dysfunction mostly related with circadian rhythms, mood, and sleep; and also with the pupillary reflex: the major mRGC functions. Morphological and connectivity studies about mRGCs have also demonstrated its relationship with dopaminergic cells, which make contacts in the S1 strata of the IPL with mRGC somas and dendrites, mainly with the M1d type [38]. Diminution of dopamine levels in the retina in PD [26] may be one of the causes of M1d cell degeneration, as this would represent a loss of one of their main synaptic inputs.

In animal models, MPTP-treated monkeys exhibit dopaminergic system impairment and circadian rhythm disruption with altered sleep/wake cycle, REM sleep impairment and daytime sleepiness [15, 23, 51, 56]. Also, in P23H blind rats, degeneration of mRGCs statistically correlates with circadian rhythms impairment [34]. Other existing works that analyzed the effect of parkinsonism in circadian rhythms described changes in the expression of the “clock genes” [12], in circadian melatonin secretion [9], in pupillary reflex [4, 54], depression [57] and in REM sleep [10, 52], all directly or indirectly controlled and affected by mRGCs. But the suprachiasmatic nucleus has been found to be unaffected until advanced stages of the disease, suggesting that there are other components of the circadian system causing circadian abnormalities in PD [22]. Thus, it is easy to question the implication of the retina, and specifically of the mRGCs, in circadian dysfunction in PD, but until now no cellular studies were available to determine its real contribution. The retinal melanopsin system abnormalities detected in PD in the present study help to explain some of the circadian and sleep problems that are common in the disease, as it probably contributes to or worsens them. The loss of mRGCs have also been described in other neurological pathologies like Alzheimer’s disease and diabetic retinopathy [31, 44] where its impairment is related to circadian rhythm alterations and sleep disorders [32, 35].

There is growing evidence that circadian rhythm disorders, normally accompanied by sleep disruption, not only negatively affect the patients’ quality of life but may also accelerate the progression of neurodegenerative disease pathology [41]. The identification and management of these symptoms is therefore important not only for a clinical benefit but perhaps also for modulation of disease progression. In this sense, knowing the effect that the retina and mRGCs may have in the progression of circadian disorders, eye protection should be recommended to patients. Additionally, novel therapies using light stimulation to synchronize circadian rhythms are demonstrating beneficial results in PD [24, 29, 36]. Martino et al. found that long-term light therapy improves sleep quality, reduces awakenings during the night and increases the total sleep time [42]. Light therapy was also found to be effective for excessive daytime sleepiness and global sleep quality [53]. A cellular explanation of this light therapy success might possibly invoke the stimulation of mRGCs, leading to dopamine release and to circadian rhythms synchronization, globally improving PD pathology [37].

## Conclusions

In summary, the present work demonstrates that the retinal melanopsin system is affected in PD. This fact has clinical implications for PD-related circadian rhythm alteration as well as for mood and sleep disorders. Protecting the retina to prolong mRGC health and using light therapies could be beneficial for the maintenance of circadian rhythms and for improving global life quality of patients.
